# Efficacy of a triple therapy for *Helicobacter pylori* eradication in a well-developed urban area in Brazil

**DOI:** 10.1590/S1516-31802004000200009

**Published:** 2004-03-01

**Authors:** Patrick Bellelis, Eliana Sueco Tibana Samano, Ricardo Cruz Nunes, Lia de Melo Ribeiro, Ethel Zimberg Chehter, Wilson Roberto Catapani

**Keywords:** *Helicobacter pylori*, Antibiotics, Therapy, Duodenal ulcer, Tratamento, Úlcera péptica, *Helicobacter pylori*, Antibióticos, Úlcera gástrica, Úlcera duodenal

## Abstract

**CONTEXT:**

*Helicobacter pylori* eradication has become the standard treatment for peptic ulcer disease. Triple therapy with omeprazole plus two antibiotics has been used. Due to the lack of ideal treatment and the high rates of primary resistance to nitroimidazoles, the use of clarithromycin has been adopted.

**OBJECTIVE:**

To determine the *Helicobacter pylori* eradication rates using lansoprazole, amoxicillin and clarithromycin for seven days, in patients with peptic ulcer disease in a well developed urban area in Brazil.

**METHODS:**

This was a retrospective, open-label study carried out at the School of Medicine of the Fundação ABC. It included 130 patients with peptic ulcer disease (upper endoscopy) who had been tested positive for *Helicobacter pylori* infection (urease test, histology or breath test), without previous treatment. Patients were treated with lansoprazole 30 mg, amoxicillin 1,000 mg and clarithromycin 500 mg b.i.d., for seven days. Eradication was verified after 90 days.

**RESULTS:**

Follow-up data were available for 94 patients. Their mean age was 52.23 years; 51.54% were woman, 84.31% white, 37.69% smokers, 20.77% using nonsteroidal anti-inflammatory drugs and 8.46% alcoholics. Upper endoscopy revealed that 78.46% had duodenal ulcers and 21.53% had gastric ulcers (a 4:1 DU:GU ratio). The eradication rates were 85.11% per protocol and 61.54% by intention to treat; 97% had no adverse effects.

**CONCLUSION:**

Triple therapyusing lansoprazole, amoxicillin and clarithromycin is well tolerated with high eradication rates and forms a good alternative for developing countries.

## RESUMO

**CONTEXTO:**

A erradicação do *Helicobacter pylori* tem sido o principal tratamento para a doença ulcerosa péptica. Consiste em uma terapia tripla com omeprazol juntamente com dois antibióticos. Devido à inexistência de uma medicação ideal, assim como a um alto índice de resistência primária aos nitroimidazólicos em nosso meio, tem-se adotado o uso da claritromicina.

**OBJETIVOS:**

Determinar o índice de erradicação do *Helicobacter pylori* em pacientes com do-ença ulcerosa péptica mediante a terapia com lanzoprazol, amoxicilina e claritromicina, por sete dias.

**MÉTODOS:**

Num estudo retrospectivo, aberto e realizado na Faculdade de Medicina do ABC, foram incluídos 130 pacientes portadores de doença ulcerosa péptica (endoscopia digestiva alta) e com infecção por *Helicobacter pylori* diagnosticada pelos testes da urease, respiratório e histológico; todos sem tratamento prévio. Os pacientes foram tratados com lanzoprazol (30 mg), amoxicilina (1.000 mg) e claritromicina (500 mg) duas vezes ao dia por sete dias. Os índices de erradicação foram avaliados noventa dias após o tratamento.

**RESULTADOS:**

94 pacientes completaram o es-tudo, sendo a média de idade 52,23 anos, 51,54% mulheres, 84,31% brancos, 37,69% tabagistas, 20,77% usuários de antiinfla-matórios não-hormonais e 8,46% de etilistas. A endoscopia digestiva alta revelou: 78,46% de úlcera duodenal (UD) e 21,53% de úlcera gástrica (UG) (4UD:1UG). O índice de erradicação por protocolo foi de 85,11% e por intenção de tratamento foi de 97%. Fo-ram observados poucos efeitos colaterais.

**CONCLUSÃO:**

A terapia tríplice com lanzoprazol, amoxicilina e claritromicina mostrou-se bem tolerável, com altos índices de erradicação, sendo uma boa alternativa para países em desenvolvimento.

## INTRODUCTION

Since the first evidence emerged in 1983, further evidence has accumulated that *Helicobacter pylori (H. pylori)* plays a major pathogenetic role in peptic ulcer disease.^1^ A meta-analysis^2^ study has shown that *H. pylori* eradication reduces peptic ulcer recurrence. Thus, *H. pylori* eradication has become the treatment of choice for curing peptic ulcer disease and preventing ulcer complications.^1^

*H. pylori* eradication is indicated for all patients with peptic ulcer, especially in cases of duodenal ulcers that are not induced by aspirin or nonsteroidal anti-inflammatory drugs (NSAIDs), including those with complications. It is also indicated in cases with low-grade gastric mucosa-associated lymphoid tissue lymphoma or atrophic gastritis, and following gastric cancer resection.^1^

One-week regimens consisting of omeprazole 20 mg b.i.d., amoxicillin 1,000 mg b.i.d. and clarithromycin 500 mg b.i.d. have been widely used in *H. pylori* eradication. However, studies with these drugs performed in some countries, including the United States and France, have had success rates ranging from 66% to 86% and from 56% to 84%, respectively.^3^

It is known that *H. pylori* develops resistance to two groups of drugs: nitroimidazoles (tinidazole and metronidazole) and macrolides (clarithromycin and azithromycine). The primary resistance of *H. pylori* strains to macrolides appears to be approximately 10%, but this may cause a significant decrease in the eradication rate.^3^

Clarithromycin seems to be an important component in eradication therapies because of the good results obtained when used on its own and in association. In Brazil, primary resistance to clarithromycin is still low, less than 5%,^3^ while in other countries it might be less than 10%.^3^

The aim of this study was therefore to retrospectively determine the *H. pylori* eradication rate with triple therapy using lansoprazole, amoxicillin and clarithromycin (LAC) for seven days, in patients with peptic ulcer disease from a well-developed urban Brazilian region (the ABC area of the São Paulo metropolitan region). The question was what kind of therapeutic pattern this region would have: whether it would be similar to well-developed countries or not.

## METHODS

This was a retrospective, open-label study carried out at the School of Medicine of the Fundação ABC. It included 130 patients with peptic ulcer disease (upper endoscopy) who had been tested positive for *H. pylori* infection (urease test, histology or breath test), without previous treatment. Patients were treated with lansoprazole 30 mg, amoxicillin 1,000 mg and clarithromycin 500 mg b.i.d. for seven days. Eradication was verified after 90 days by means of the urease test, breath test or upper endoscopy.

### Statistical analysis

The statistical analysis was performed using the NCSS-2000 software (Utah, United States) for descriptive analysis of frequencies. For correlations between discrete variables we used the chi-squared test and Fisher's exact test. We also utilized variance analysis between the eradicated, non-eradicated and dropout groups. p < 0.05 was considered significant throughout the study.

## RESULTS

Follow-up data were available for 94 patients. Sixty-seven were female (51.54%) and the mean age was 52.23 years. Tobacco and NSAIDs users made up a small number in the group, as did alcoholic patients. The majority were born in Brazil's southeastern region (62.31%) and came from the ABC region (82.32%) (Table 1).

**Table 1:** Baseline data in peptic ulcer disease population

	n	%
Women	67	51.54
Men	63	48.46
White	107	82.31
Southeastern region	81	62.31
ABC region	107	82.32
Tobacco users	49	37.69
Alcohol users	11	8.46
NSAIDs users	27	20.77

*n = number; NSAIDs: nonsteroidal anti-inflammatory drugs.*

There were nearly four times more duodenal ulcers than gastric ulcers. The *H. pylori* eradication rates were 85.11% by per protocol analysis and 61.54% by intention to treat analysis. The primary endoscopy found 117 scarred ulcers and 13 active ulcers, all of which were positive for *H. pylori* infection. Upper endoscopy revealed that 78.46% of the patients had duodenal ulcers and 21.53% had gastric ulcers (a 4:1 DU:GU ratio). The active ulcers were duodenal, and therefore upper endoscopy after treatment was not available: only the breath test was (Table 2).

**Table 1 2.** Main results in peptic ulcer eradication in the ABC region

	n	%
Gastric ulcers	28	21.53
Duodenal ulcers	102	78.46
DU:GU	102:28	3.64:1
Control after treatment	94	72.31
Adverse events	3	2.31
Eradication *per protocol*	80	85.11
Eradication by intention to treat	80	61.54

*n = number; DU: duodenal ulcer; GU: gastric ulcer.*

The treatment compliance was 72.31%, with low rates (3%) of adverse effects.

### Statistical analysis

No statistically significant correlations could be found between eradication and the use of tobacco, alcohol or NSAIDs, or between eradication and demographic data such as age, gender and race. Variance analysis did not find a significant statistical correlation between the eradicated, non-eradicated and dropout groups, for age.

## DISCUSSION

The ABC region consists of seven cities (Diadema, Mauá, Ribeirão Pires, Rio Grande da Serra, Santo André, São Bernardo do Campo and São Caetano do Sul) in the São Paulo Metropolitan Region, with 2.5 million inhabitants. This is one of the most industrialized areas of Brazil and it has a *per capita* income of U$ 3,250.00, thereby making this the third biggest market in Brazil, after São Paulo and Rio de Janeiro.^4^

Santo André and São Bernardo do Campo have the best Human Development Index (HDI) (0.813 and 0.808 respectively) in this region.^4^ About 83% of the patients studied were from these cities, and this population was thus characterized as having a higher socioeconomic level than the Brazilian population as a whole. The majority of the population studied consisted of white people and were not tobacco, alcohol or NSAIDs users.

The male-female ratio in the ABC region is approximately 1:1 (51% women), which is similar to what was found in our study. This result differs from what was found in a French population, where there was male predominance (2:1).^3^ However, this difference did not significantly affect the eradication rates, nor did the age and tobacco use.^1^

The mean age of our patients was 52.2, which is a little older than what was found in other studies (44 to 50 years).^3^ Theoretically, this could represent late infection, due to the better living conditions of our population.

The eradication rates from triple therapy have been found to vary from center to center around the world.^5^ The reason for such differences is not clear, but many factors seem to be involved. Our study found eradication rates that were similar to the rates in another center, which ranged from 78% up to 90% *per protocol*, using 10-day schedules.^5^ Our intention-to-treat group had a lower eradication rate (61.54%) than for studies using 10 days of treatment (69% to 83%).^5^ This low eradication rate in the intention-to-treat group could be explained by our high dropout rate (27.7%). We believe that this high dropout was due to the improvement of patients' clinical symptoms.

However, because of the high nitroimidazole resistance rate in Brazil, a search for new therapeutic schedules is necessary.

Although triple therapy based on lansoprazole, amoxicillin and clarithromycin is more expensive than bismuth, metronidazole and tetracycline, its use is justified by the high nitroimidazole resistance rate among our population. It is also convenient to use, since it is sold in blister packs.

## CONCLUSION

Triple therapy using lansoprazole, amoxicillin and clarithromycin is well tolerated, shows high eradication rates and is a good alternative for developing countries.
